# Supplementary material for: Nuclear Physics Meets the Sources of the Ultra-High Energy Cosmic Rays
Source: Sci Rep. 2017 Jul 7;7:4882. doi: 10.1038/s41598-017-05120-7 (PMC5501817; doi:10.1038/s41598-017-05120-7)
Supplement: Supplementary file 1 — Supplementary Material [file 41598_2017_5120_MOESM1_ESM.pdf]

SUPPLEMENTARY MATERIAL
Nuclear Physics Meets the Sources of the Ultra-High Energy Cosmic Rays

Denise Boncioli, Anatoli Fedynitch, and Walter Winter
DESY, Platanenallee 6, D-15738 Zeuthen, Germany
(Dated: May 4, 2017)

I. CROSS SECTION TERMINOLOGY AND COMPUTATION OF DISINTEGRATION LENGTHS

Numerical solutions of our system of Boltzmann transport equations (see the Methods section in the main text) require knowledge about the rate of interactions and the average outcome of the bulk of all possible reactions. The interaction rate Γ_j of the primary nucleus j is used to compute the secondary injection and primary escape flux. The interaction rate depends on the interaction partner, the photon, as

$$\Gamma_j(E_j) \; = \; \int d\varepsilon \int\limits_{-1}^{+1} \frac{d\cos\theta_{j\gamma}}{2} \, (1-\cos\theta_{j\gamma}) n_\gamma(\varepsilon, \cos\theta_{j\gamma}) \, \sigma_j^{\mathrm{abs}}(\epsilon_r) \, . \tag{A}$$

Here $n_\gamma(\varepsilon, \cos\theta_{j\gamma})$ is the photon density as a function of photon energy ε and the (pitch) angle between the photon and proton momenta $\theta_{j\gamma}$, $\sigma_j^{\mathrm{abs}}(\epsilon_r)$ is the absorption cross section and

$$\epsilon_r = \frac{E_j \, \varepsilon}{m_j} (1-\cos\theta_{j\gamma}) \tag{B}$$

is the photon energy in the parent rest frame (PRF) or nucleus rest frame in the limit $\beta_A \approx 1$, and can be directly related to the available center-of-mass energy. The interaction itself, and therefore E_j and ε, is typically defined in the shock rest frame (SRF). Eq. (B) clearly shows that the relevant energy range in the PRF is defined by the product of the target photon's energy times the nucleus energy in the SRF and is therefore highly dependent on both, the choice of the target photon and the injection spectrum. In our models we assume isotropy $n_\gamma(\varepsilon, \cos\theta_{p\gamma}) \simeq n_\gamma(\varepsilon)$ of the target photon distribution. This means that, for GRBs, we compute the interactions in the shock rest frame where isotropy occurs if the target photons come from self-consistent internal radiation processes (such as synchrotron emission of electrons/positrons). Isotropy smears out the substructure of the energy dependence of σ_{abs}.

The injection $j \to i$ of secondaries Q_{ji}, which we need in Eq. 2 (Methods section), is given by

$$Q_{ji}(E_i) = \int dE_j \, N_j(E_j) \, \frac{d\Gamma_{ji}(E_j)}{dE_i} \, , \tag{C}$$

where $d\Gamma_{ji}(E_j)/dE_i$ can be obtained from Eq. (A) by replacing the total absorption cross section with the differential inclusive cross section

$$\sigma_j^{\mathrm{abs}}(\epsilon_r) \to \frac{d\sigma_{j\to i}^{\mathrm{incl}}}{dE_i} \equiv \sigma_j^{\mathrm{abs}}(\epsilon_r) \, \frac{dn_{j\to i}}{dE_i} \, , \tag{D}$$

where $dn_{j\to i}/dE_i$ is the re-distribution function of the secondaries. For the energy spectrum of secondaries, we make use of typical kinematics, where for example, for GRBs, the nuclei have PeV energies and the photons are in keV range. In that case the momentum transfer on the ejected secondaries is small compared to E_j, resulting in an approximate conservation of the relativistic boost. In that case, the re-distribution function can be approximated as a function of ϵ_r and E_i/E_j as

$$\frac{dn_{j\to i}}{dE_i} \simeq M_{j\to i}(\epsilon_r) \cdot \delta\left(E_i - \frac{m_i}{m_j} E_j\right) \, , \tag{E}$$

where $M_{j\to i}$ is the average number of secondaries produced per interaction (also called "multiplicity"). It can be related to the exclusive reaction cross sections, summed over all channels in which the species i is produced, as

$$M_{j\to i} = \frac{1}{\sigma_j^{\mathrm{abs}}} \sum_{X_n} N_i \, \sigma_j^{\mathrm{excl}}(\gamma, X_n + i) \, , \tag{F}$$

where N_i is the number of secondaries i in that exclusive channel. Note that the ratio between exclusive and total absorption cross section corresponds to the branching ratio. While for light fragments, M can be larger than one, one expects for residual nuclei $M \lesssim 1$.

Finally, note that fixed target experiments in photon beams typically measure the cross section in terms of PRF energy, and it is a frequently asked question up to which energy the cross section needs to be measured. In astrophysical environments, a certain PRF energy can be related to the primary E_j and photon ε energies by the estimate $\epsilon_r \sim E_j\,\varepsilon/m_j$, neglecting the pitch angle averaging, see Eq. (B). Astrophysical photon spectra typically exhibit features, such as a spectral break (such as for GRBs) or a maximum (such as for the CMB) at a certain $\hat{\varepsilon}$, whereas the cosmic ray primaries typically follow a power law. As a consequence, given a cross section $\sigma(\epsilon_r)$ at a certain energy ϵ_r, primaries with the energy $E_j \simeq \epsilon_r m_j/\hat{\varepsilon}$ will be selected to interact, and the secondary spectra will exhibit spectral excesses corresponding to that primary energy. In that sense, $\sigma(\epsilon_r)$ as a function of the PRF is the required input for astrophysical applications, and a definition of a target photon spectrum in the nucleus' rest frame does not make sense. Larger values of ϵ_r just mean that the spectral features will appear at higher energies in E_j if the cross section is significantly large there. For all practical cases, one therefore needs the cross section in the region around the GDR up to the point where it significantly drops. This can be seen *e.g.* in Fig. 1 by comparing the PEANUT cross section curve with the measurement for ^{23}Na, which are practically identical up to the maximally measured energy. The difference in the disintegration length is nevertheless large, as the contribution beyond $\epsilon_r \simeq 30$ MeV, which is missing in the measurement case, cannot be neglected (and will be re-distributed by the pitch angle averaging). The direct correlation $E_j \propto \epsilon_r$, translating cross section peaks into the disintegration rate, can be best seen in the CMB cases in Fig. 1.

II. SITUATION ON EXPERIMENTAL DATA AND MODEL CALCULATIONS

Here we summarize the status on experimental data and theoretical models.

A. Experimental data

EXFOR [1] is a nuclear reaction database, aimed to be a complete collection of experiments and theoretical evaluations performed during the last half century. The effort to maintain and standardize the data collection is a world-wide collaborative effort between various nuclear centers. In this work, we assume that this database is to a large extent complete and our result is not distorted by one or few missing entries.

The database contains experimental data points, model-evaluated data, ratios of cross sections and other data categories. For our purpose we apply the following criteria to the data selection:

- only real experimental data, no evaluated cross sections;
- no reaction combinations, ratios or partial measurements;
- full unfolded cross sections in barns;
- energy range of the measurement has to cover the GDR peak.

Further, we create two selections (EXFOR, $\sigma_{\rm abs}$) and (EXFOR, any), where the first includes only measured absorption cross sections $\sigma_{\rm abs}$ and the latter requires at least one inclusive cross section to be measured. With these criteria, EXFOR contains 14 absolute cross sections and 47 of the second category up to masses of ^{56}Fe. (EXFOR, any) can be used to estimate the number of more certain absorption cross sections from nuclear reaction models.

B. Theoretical models

Monte Carlo particle transport codes, such as FLUKA [2, 3], MCNP [4] or PHITS [5] use evaluated nuclear data files to compute interaction rates and Intranuclear Cascade Pre-equilibrium Evaporation (ICPE) codes for exclusive reaction cross/sections. FLUKA contains a comprehensive photo-nuclear cross section library based direct on data, photo-neutron evaluation and parameterizations for a large set of isotopes [6, 7]. It is coupled to the ICPE code PEANUT to generate final state distributions. Comparisons of PEANUT cross sections reveal a very good agreement to available data on $\sigma_{\rm abs}$. MCNP employs in a similar manner ENDF-B-VII.1 [8] as evaluated nuclear data library, based on reaction model calculations using the GNASH code system. For photo-nuclear data file for PHITS is

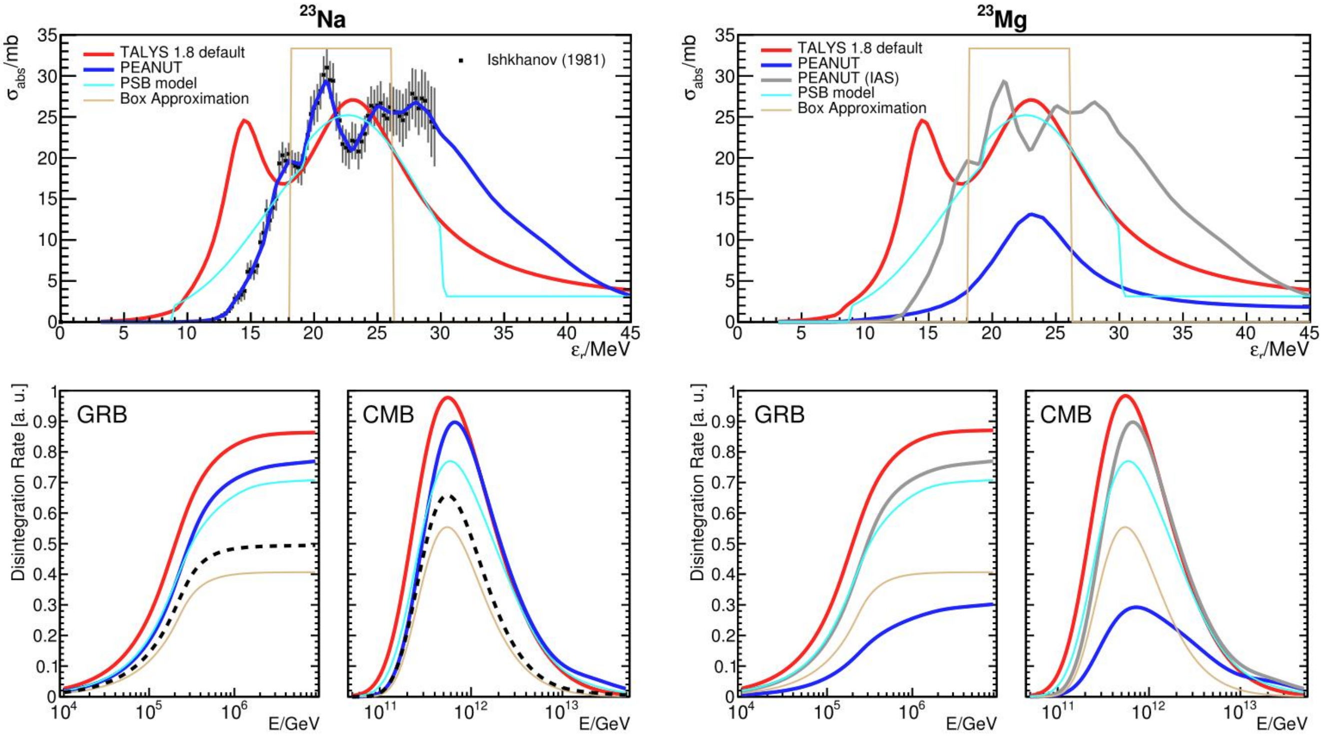

Supplementary Figure 1: Same as in Fig. 2 (main text), for $A = 23$.

JENDL/PD-2004 [9], which has often good agreement with data but also shows some interpolation or fitting artifacts for light and intermediate nuclei.

Our current baseline model is based on cross sections extracted from TALYS 1.8 for nuclei with $A \geq 12$ and CRPropa2 lighter nuclei. TALYS 1.8 was configured to use the Kopecki-Uhl generalized Lorentzian description, as it is recommended in [10] in order to better predict cross sections not only in the GDR region. Photo-disintegration cross sections for light nuclei in CRPropa2 [11] are compiled from various references as follows: ^{9}Be, ^{4}He, ^{3}He, ^{3}H, and ^{2}H as given in [12]; ^{8}Li, ^{9}Li, ^{7}Be, ^{10}Be, ^{11}Be, ^{8}B, ^{10}B, ^{11}B, ^{9}C, ^{10}C and ^{11}C as given in [13]; ^{7}Li as given in [14, 15]. In total we have from CRPropa2 19 primary isotopes and 69 inclusive cross sections. The TALYS 1.6 cross sections are currently used in CRPropa3, to predict photo-disintegration products of all available exclusive channels: proton, neutron, ^{2}H, ^{3}H, ^{3}He, ^{4}He and combinations thereof; the parameters used to model the giant dipole resonance have been adjusted to match the cross sections reported in [10], as explained in [16]. The *SimProp* code uses an approximation scheme to implement TALYS cross sections, as reported in [17].

The PSB model [18], which is tailored for cosmic ray propagation problems, represents each isobar with the most abundant stable isotope, starting from ^{56}Fe down to ^{2}H, excluding the unstable masses $5 \leq A \leq 8$. This model approximates the cross sections for one- and two-nucleon emission with a Gaussian shape in the low energy range ($2 \leq \epsilon_r \leq 30$ MeV), while the cross sections for multi-nucleon emission in the high energy range ($30 \leq \epsilon_r \leq 150$ MeV) are constant. In *SimProp* the list of isobars matches [18], but the corresponding elements are assigned from Table 1 in [19] by choosing the isotope that corresponds to the lowest energy threshold for the emission of one proton. For the purpose of the present work, we have slightly changed the criterion for choosing the element with respect to what has been done in *SimProp* or in the original PSB model, that allowed for example for $A \rightarrow A' = A - 1$ with an increasing number of protons with respect to the parent nucleus, by choosing descending elements in the chain corresponding to the mass in the original list.

III. DISINTEGRATION RATES FOR ALTERNATIVE ELEMENTS

To illustrate a second example in addition to Fig. 2 (main text), we compare cross sections of ^{23}Na and ^{23}Mg in Fig. 1. Contrary to the double-magic ^{40}Ca, ^{23}Na is not expected to be spherical, where GDR cross sections can be sufficiently approximated using a single peak. Therefore, the models with only one main Lorentzian or Gaussian

peak do not reproduce the measurement, as it is the case for the PSB model. Without dedicated Lorentzian fits, TALYS doesn't reproduce the shape of the GDR peak, adding an additional, unphysical, peak at ~ 14 MeV. The latter can be attributed to the strength parameter setting and can be potentially corrected through individual tuning. However, such modification would alter the model for a single isotope without improving the predictive power for other isobars. The PEANUT prediction reproduces very well the available measurement [20] on ^{23}Na, while the box model is insufficient and yields factor of two smaller disintegration rates. For the unknown ^{23}Mg cross section, TALYS, PSB and the box model predict identical cross sections, while PEANUT falls back to a parametrized form, producing a significantly different result. Updated versions of PEANUT and FLUKA[1] estimate unknown cross sections with the Isobaric Analog State (IAS) approach, where nuclei with identical wave functions, and therefore, with a similar isospin and level structure are treated analogously. If such an equivalence case is detected, the model maps unknown cross sections of nuclei to a known IAS nucleus. The effect of IAS is illustrated in the right panel Fig. 1 and results in up to a factor three higher disintegration rates in the case of PEANUT. If IAS is a valid concept for photon projectiles, several additional cross sections can become known to higher precision without the need for individual measurements.

[1] Otuka, N. *et al.* Towards a More Complete and Accurate Experimental Nuclear Reaction Data Library (EXFOR): International Collaboration Between Nuclear Reaction Data Centres (NRDC). *Nuclear Data Sheets* **120**, 272 – 276 (2014). URL `http://www.sciencedirect.com/science/article/pii/S0090375214005171`.

[2] Ferrari, A., Sala, P. R., Fasso, A. & Ranft, J. FLUKA: A multi-particle transport code (Program version 2005). *CERN-2005-010* (2005).

[3] Böhlen, T. *et al.* The FLUKA Code: Developments and Challenges for High Energy and Medical Applications. *Nuclear Data Sheets* **120**, 211 – 214 (2014). URL `http://www.sciencedirect.com/science/article/pii/S0090375214005018`.

[4] Goorley, J. T. *et al.* Initial MCNP6 Release Overview - MCNP6 version 1.0. Tech. Rep. LA-UR-13-22934 (2013).

[5] Sato, T. *et al.* Particle and Heavy Ion Transport code System, PHITS, version 2.52. *Journal of Nuclear Science and Technology* **50**, 913–923 (2013). URL `http://dx.doi.org/10.1080/00223131.2013.814553`.

[6] Fassò, A., Ferrari, A. & Sala, P. R. Total Giant Resonance Photonuclear Cross Sections For Light Nuclei. *Proc. SATIF-3, Sendai, Japan* 61–74 (1997).

[7] Fassò, A., Ferrari, A. & Sala, P. R. Photonuclear Reactions in FLUKA: Cross Sections and Interaction Models. *AIP Conference Proceedings* **769**, 1303–1306 (2005). URL `http://scitation.aip.org/content/aip/proceeding/aipcp/10.1063/1.1945245`.

[8] Chadwick, M. *et al.* ENDF/B-VII.1 Nuclear Data for Science and Technology: Cross Sections, Covariances, Fission Product Yields and Decay Data. *Nuclear Data Sheets* **112**, 2887 – 2996 (2011). URL `http://www.sciencedirect.com/science/article/pii/S009037521100113X`, special Issue on ENDF/B-VII.1 Library.

[9] Kishida, N. *et al.* JENDL Photonuclear Data File. *AIP Conference Proceedings* **769**, 199–202 (2005). URL `http://scitation.aip.org/content/aip/proceeding/aipcp/10.1063/1.1944989`.

[10] Khan, E. *et al.* Photodisintegration of ultra-high-energy cosmic rays revisited. *Astropart. Phys.* **23**, 191–201 (2005).

[11] Kampert, K.-H. *et al.* CRPropa 2.0 – a Public Framework for Propagating High Energy Nuclei, Secondary Gamma Rays and Neutrinos. *Astropart. Phys.* **42**, 41–51 (2013).

[12] Rachen, J. P. *Interaction Processes and Statistical Properties of the Propagation of Cosmic Rays in Photon Backgrounds*. Ph.D. thesis, MPIfR Bonn, Germany (1996).

[13] Kossov, M. V. Approximation of photonuclear interaction cross-sections. *Eur. Phys. J.* **A14**, 377–392 (2002).

[14] Varlamov, V., Surgutanov, V., Chernyaev, A. & Efimkin, N. Photodisintegration of Lithium. Evaluated cross sections of channels and reactions. *Fotoyad.Dannye* (1986).

[15] Kulchitskii, L., Volkov, Y. & Denisov, V.P. Ogurtsov, V. Photodisintegration of Li^7. *Izv. Rossiiskoi Akademii Nauk, Ser Fiz.* **27**, 1412 (1963).

[16] Batista, R. A. *et al.* CRPropa 3 - a Public Astrophysical Simulation Framework for Propagating Extraterrestrial Ultra-High Energy Particles. *JCAP* **1605**, 038 (2016).

[17] Aloisio, R. *et al.* SimProp v2r3: Monte Carlo simulation code of UHECR propagation (2016).

[18] Puget, J. L., Stecker, F. W. & Bredekamp, J. H. Photonuclear Interactions of Ultrahigh-Energy Cosmic Rays and their Astrophysical Consequences. *Astrophys. J.* **205**, 638–654 (1976).

[19] Stecker, F. W. & Salamon, M. H. Photodisintegration of ultrahigh-energy cosmic rays: A New determination. *Astrophys. J.* **512**, 521–526 (1999).

[20] Ishkhanov, B. S., Kapitonov, I. M., Shvedunov, V. I., Gutiy, A. I. & Parlag, A. M. Investigation of the reaction $^{23}Na(\gamma,p)^{22}Na$ with production of the final nucleus in various states. *Yadernaya Fizika* **33**, 581 (1981).

[1] Private communication with Alfredo Ferrari (07/2016)
